# Research progress on the Sonic Hedgehog signaling pathway in the central nervous system: Novel insights

**DOI:** 10.4103/NRR.NRR-D-24-01239

**Published:** 2025-08-13

**Authors:** Nuokun Li, Shiyi Wen, Dandan Li, Yaning Shi, Zhigang Mei, Danhong Liu, Hui Yang, Yuhong Wang, Xiaoyuan Lin, Yun Xiang, Hongbo Wen, Pan Meng

**Affiliations:** 1 Science & Technology Innovation Center, Hunan University of Chinese Medicine, Changsha, Hunan Province, China; 2 Department of Plastic Surgery, Third Xiangya Hospital, Central South University, Changsha, Hunan Province, China; 3 Department of Science and Technology, Hunan University of Chinese Medicine, Changsha, Hunan Province, China; 4 School of Integrated Chinese and Western Medicine, Hunan University of Chinese Medicine, Changsha, Hunan Province, China; 5 The First Hospital, Hunan University of Chinese Medicine, Changsha, Hunan Province, China; 6 Department of Neurosurgery, Yiyang Central Hospital of Hunan Province, Yiyang, Hunan Province, China

**Keywords:** Alzheimer’s disease, canonical, central nervous system disease, drug therapy, neural regeneration, non-canonical, Sonic Hedgehog, Sonic Hedgehog medulloblastoma, stroke

## Abstract

Over the past few decades, the Sonic Hedgehog protein has become a pivotal player in many biological processes, including tumourigenesis, embryonic development, and protective mechanisms after cerebral damage. The Sonic Hedgehog signaling pathway is crucial in the central nervous system, with implications in a diverse range of diseases, including Parkinson’s disease, Alzheimer’s disease, spinal cord injury, traumatic brain injury, depression, Sonic Hedgehog medulloblastoma, and stroke. In this comprehensive review, we examined Sonic Hedgehog from the perspective of canonical and non-canonical pathways, elucidating their complex connections to the central nervous system. Subsequently, we summarize the latest advancements in drug therapies that offer novel strategies for treating neurological diseases by modulating the Sonic Hedgehog protein. Finally, we summarize and extend the technologies and tools for studying the Sonic Hedgehog signaling field, with the aim of providing new research ideas and methods.

## Introduction

The central nervous system (CNS) is derived from the ectoderm of germ disks. During the neural plate stage of embryogenesis, the notochord acts as an axial structure running through the embryo during early development, inducing the transformation of undifferentiated ectodermal cells above it into CNS precursors (Arendt et al., 2008). Initially, the dorsal ectodermal cells above the notochord elongate and thicken, forming a neural plate with a broad anterior and narrow posterior. The edges of the neural plate thicken and fold to form neural folds, while the center of the neural plate depresses to create a neural groove (Jayachandran et al., 2016). As the neural folds gradually merge and fuse, a hollow neural tube forms and detaches from the ectoderm. During development, neurons detach from the ectoderm and migrate inward to occupy the brain and spinal cord, forming the main components of the CNS (Karkali et al., 2023). The Sonic Hedgehog (SHH) expression has been detected in the mesoderm and neural tubes (Alaynick et al., 2011; Blaess et al., 2014).

The expression products of the Hedgehog (HH) gene family are a series of secretory proteins that can act on intracellular and distant cells to mediate relevant gene expression levels (Yang et al., 2021). In mammals, desert hedgehog, Indian hedgehog, and SHH are homologous HH family members (Geyer and Gerling, 2021). Indian HH regulates endochondral ossification as a regulator of bone development, which couples chondrogenesis to osteogenesis during endochondral bone development (Chung et al., 2001). Desert HH plays an important role in the development of gonadal tissue (Sigafoos et al., 2021), and desert HH signaling is essential in regulating mammalian spermatogenesis (Bitgood et al., 1996). Of the three types of *HH* genes, the *SHH* gene was first identified in a wingless mutation of Drosophila (Nüsslein-Volhard and Wieschaus, 1980). It is widely expressed in human tissues and is involved in the development of limbs, skin, hair, cochlea, axial bone, lungs, brain, and spinal cord (Echevarría-Andino and Allen, 2020). SHH, Patched1 (PTCH1), Smoothed (SMO), and glioma-associated oncogene (GLI) are the core proteins of the SHH signaling pathway. In addition, a series of highly coordinated ligands, receptors, cytoplasmic signaling molecules, transcription factors, and co-regulators regulate biological functions controlled by this pathway (Maschinot et al., 2015). SHH signaling pathway–related mechanisms are complex, with cross-talk occurring among various pathways and proteins sharing the same target protein, making SHH function extensive. Hence, this article summarises the mechanism of SHH and its signaling pathways (including non-canonical signaling pathways), their deep connection with the CNS, the impact of SHH on CNS diseases, and its therapeutic effects.

## Literature Search Strategies

**Figure 1:**
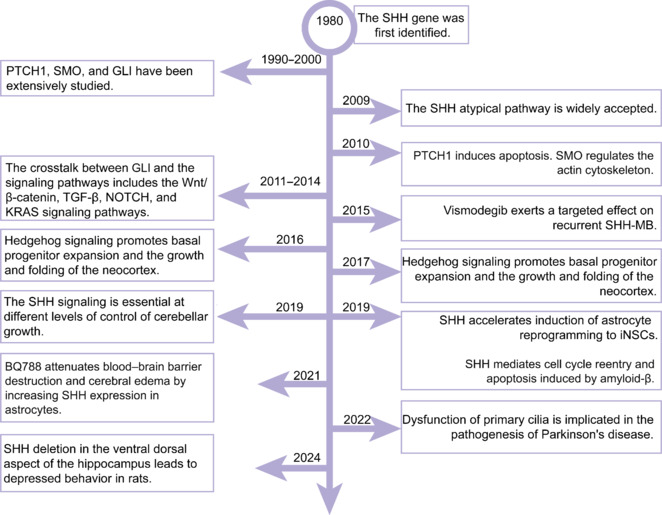
Chronological events in the study of the SHH signaling pathway in the central nervous system. GLI: Glioma-associated oncogene; iNSC: islet neuroendocrine stem cells; PTCH1: Patched1; SHH: Sonic Hedgehog; SHH-MB: Sonic Hedgehog-medulloblastoma; SMO: Smoothed; TGF-β: transforming growth factor-β.

In this comprehensive review, articles published in PubMed up to the year 2025 were searched. The literature was searched using the following keywords: central nervous system, SHH, PTCH1, SMO, GLI, canonical hedgehog signaling pathway, non-canonical hedgehog signaling pathway, WNT, TGF-β, NOTCH, KRAS, neuronal proliferation and differentiation, axonal guidance, neurogenesis, astrocytes, neurons, mitochondrial abnormalities, neurodegenerative diseases, depression, Parkinson’s disease, Alzheimer’s disease, spinal cord injury, traumatic brain injury, Sonic Hedgehog medulloblastoma, treatment, chemical drugs, traditional Chinese medicine, exosomes, non-coding RNA, stroke, brain tumor, stem cell therapy, neuroinflammation, gene editing, computational biology, bioinformatics. We removed irrelevant and duplicate studies from the retrieved research, eliminated articles that were not highly relevant or deemed unnecessary based on their titles and abstracts, and then, read the full text and included studies that provided a clear explanation of sample size, experimental methods, and procedures. Chronological events in the study of the SHH signaling pathway in the central nervous system are shown in **Figure 1**.

## Sonic Hedgehog Signaling Pathway

### Sonic Hedgehog canonical signaling pathway

All three mammalian HH ligands exhibit high sequence homology and are the initial ligands that trigger HH signaling transduction. The inaugural step of the signaling cascade involves the N-terminal cleavage of approximately 25 amino acids from the 46 kDa precursor molecule, removing the signal peptide (Sigafoos et al., 2021). The remaining protein undergoes an autocatalytic cleavage reaction, yielding a 19 kDa N-terminal fragment (HH-N) and a 25 kDa C-terminal fragment (HH-C). This cleavage process is mediated by proteolytic mechanisms within the endoplasmic reticulum (ER) (Porter et al., 1996). The HH-C fragment possesses catalytic activity and is responsible for the covalent attachment of a cholesterol (CHOL) molecule to the C-terminus of HH-N, after which HH-C is ubiquitinated and degraded by the proteasome (Li et al., 2021b). Furthermore, the N-terminal domain of HH-N undergoes palmitoylation, catalyzed by an HH acyltransferase enzyme (Tang et al., 2022). Dilipidised HH-N is a completely activated signaling molecule but is still not secreted by cells. Dispatched 1, a membrane protein, has changed this with the condition (Qi and Li, 2020). In the extracellular region, other proteins and enzymes facilitate the breakdown and transfer of the SHH complex. Signal peptide, CUB domain, EGF-like 2 (Scube2) promotes SHH shedding through its epidermal growth factor and CUB domains (Ehring and Grobe, 2021). Subsequently, CAM-related/downregulated by oncogenes and brother of CAM-related/downregulated by oncogenes recruit the Scube2-SHH complex to target cells (Wierbowski et al., 2020). Growth-arrest-specific-1 receives SHH signals by binding to the Scube2-SHH complex through its carboxy-terminal tail and relays the signal to PTCH1 (Huang et al., 2022b).

PTCH1 consists of 1447 residues and includes 12 transmembrane helices (five of which include a sterol-sensing domain) and engages in interactions with the CHOL-mediated modification of SHH (Qi and Li, 2020). Notably, the fully modified SHH is highly asymmetrical, with an extended N-terminal palmitic arm and a globular Ca^2+^-binding domain. This complex binds to PTCH1 to form a repressive complex (Schonbrun and Resh, 2022), and copurifying the receptor-ligand complex showed that the inhibited receptor exists as a 2 PTCH1:1 SHH complex. Specifically, the complex is bridged to the two-lipid SHH, which binds to the PTCH1-A monomer through its globular calcium-mediated interface and binds to PTCH1-B through an extension arm formed by most N-terminal residues and N-terminal palmitate (Rudolf et al., 2019). However, in the monomer system (Petrov et al., 2020), the attachment of CHOL to the sterol-sensing domain exhibits better stability and endurance than the dimer-SHH configuration. This observation hints at a mechanism where SHH-mediated dimerization potentially frees up CHOLs sequestered by PTCH1 monomers, thereby augmenting the pool of CHOLs accessible within the cell membrane. Additionally, SHH exerts its regulatory effect on PTCH1 by modulating the availability of these accessible CHOLs (Zhong and Wang, 2022). Once bound to SHH, the receptor undergoes subsequent trafficking towards the lysosomes, where it undergoes degradation, effectively mitigating its inhibitory influence on SMO (Krausert et al., 2022).

SMO is a G protein-coupled receptor protein (Wang et al., 2022e) that possesses two CHOL-binding sites: one in the extracellular cysteine-rich domain (CRD) and the other within the transmembrane domain. Sterols function as SHH-modulated orthosteric ligands at the CRD and allosteric modulators at the transmembrane domain, thereby controlling SMO activity and SHH signaling (Kinnebrew et al., 2019). PTCH1 inhibits the binding of sterols to CRD and affects the content of sterols in and outside the cell through its transport activity (Kinnebrew et al., 2022). Together, SHH and PTCH1 regulate the CHOL modification of ER-localised SMO, facilitating its release from the ER (Qiu et al., 2023). Subsequently, SMO enters the primary cilia (PC) and regulates GLI proteins with the assistance of various proteins.

PC consists of a basal body (a specially modified centriole), ciliary transition zone, PC membrane, and axoneme (Mill et al., 2023). Numerous components of the SHH signaling pathway are dynamically shuttled into and out of the cilia in response to ligand binding. The activation of SMO causes it to accumulate in PC (Xu et al., 2020). Owing to the longer residence of SMO in the PC, the GLI-suppressor of fused (SUFU) complex within the PC dissociates, ultimately bypassing the proteolytic process (Sigulinsky et al., 2021). In other words, GLI enters the nucleus in full-length form.

**Figure 2:**
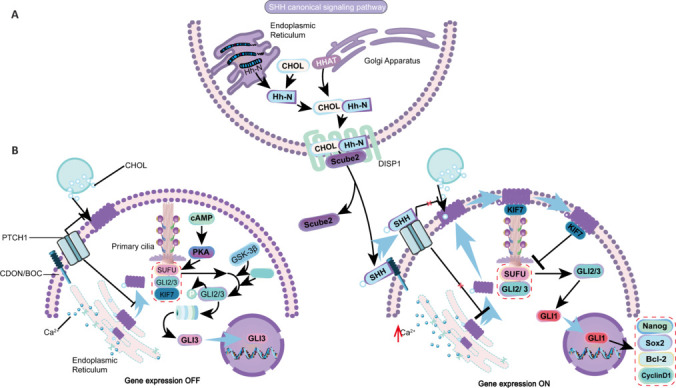
SHH canonical signaling pathway. (A) Production and transportation of HH proteins. The precursor of HH is self-cleaved, and the HH-N is covalently modified with CLR at the C-terminus. HH-N is then further palmitoylated by HHAT on the ER membrane. After these processes, it binds to the membrane protein DISP1 on the cell surface and releases HH-N into the extracellular space. The Scube2 protein assists in the transport of HH-N in the extracellular space. (B) In the absence of SHH signaling, PTCH1 inhibits the binding of cholesterol to SMO and its surface localization, and GLI protein is phosphorylated by PKA, GSK-3β, and casein kinase 1 protein kinases, cutting off the N-terminal, mainly in the form of GLI3, inhibiting the transcription of SHH pathway genes. In the presence of SHH signaling, the binding of SHH to PTCH1 relieves the inhibition of SMO by PTCH1, resulting in the re-localization of SMO to cilia. Simultaneously, cholesterol binds to SMO, activating SMO. The activated SMO forms a complex with KIF7, which can avoid GLI phosphorylation by proteases, thereby retaining the full length and inducing GLI1. It initiates downstream signaling events and activation of Gli proteins and increases the expression of SHH pathway genes. Bcl-2: B-cell CLL/lymphoma 2; cAMP: cyclic adenosine monophosphate; CDON/BOC: CAM-related/downregulated by oncogenes/brother of CDON; CHOL: cholesterol; CK1: casein kinase 1; Disp1: dispatched RND transporter family member 1; GLI: glioma-associated oncogene; GSK-3β: glycogen synthase kinase 3β; HHAT: hedgehog acyltransferase; HH-N: N-terminal fragment; KIF7: kinesin family member 7; PKA: protein kinase A; PTCH1: patched1; Scube2: signal peptide-CUB-EGF like domain-containing protein 2; SHH: Sonic Hedgehog; Sox2: SRY-box transcription factor 2; SUFU: suppressor of fused.

GLI proteins perform two diametrically opposed functions in biological reactions: activation and inhibition. GLI proteins are divided into three isoforms: GLI1, GLI2, and GLI3 (Kinzler et al., 1988). GLI1–3 contains an activation domain at the C-terminus, while only GLI2/3 contains an N-terminal suppressor domain (Ruiz i Altaba, 1999), contributing to their activity as transcriptional repressors. SUFU is associated with GLI2/3 in the signal transduction of GLI. In the absence of SHH ligands, protein kinase A phosphorylates them, which allows casein kinase 1 and glycogen synthase kinase 3β to recognize and further phosphorylate these proteins (Tay et al., 2019). Subsequently, the C-terminus of GLI2/3 is cleaved, removing the activation domain and promoting its transformation into a repressor protein form (Zhou et al., 2022). However, in the presence of a SHH ligand-activating pathway, the protein complex is not phosphorylated, thus maintaining its full length and promoting the transcription of downstream genes (Liu et al., 2023). Because GLI3 is susceptible to the regulation of proteolysis, it generally exists as a repressor (Li et al., 2004). GLI1 is not primarily regulated by proteasomal degradation but can induce the formation of GLI1 after GLI2/3 is retained in its full-length form (Sternfeld et al., 2020). Finally, it translocates to the nucleus, promoting the transcription of SHH/GLI target genes (**Figure 2**).

### Sonic Hedgehog non-canonical signaling pathway

In the SHH signaling pathway, principal proteins such as PTCH1 and SMO may be modulated not only by upstream and downstream components but also by additional proteins, cytokines, and pharmacological agents. Furthermore, alternative signaling cascades converge on the same targets as GLI transcription factors or directly intersect with the GLI pathway through cross-talk mechanisms. This distinctiveness sets it apart from the canonical SHH pathway, designated as the SHH non-canonical signaling pathway. This dichotomy facilitates a more nuanced exploration of SHH signaling modalities and enables the finer-grained regulation of diverse SHH signaling pathways. Atypical SHH signaling pathways are broadly categorized into three types: type I, type II, and type III (Jenkins, 2009; Campbell and Copland, 2015).

#### Patched-mediated signaling

**Figure 3:**
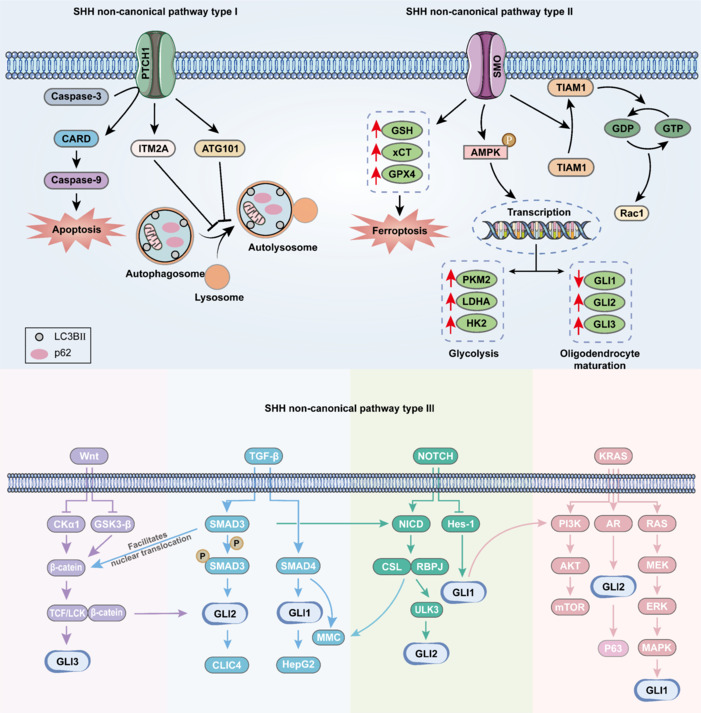
SHH non-canonical signaling pathways. Upper left: SHH non-canonical signaling pathway type I. In response to caspase-3 activation, PTCH1 is recruited and cleaved, releasing CARD, which further triggers the activation of caspase-9, ultimately culminating in apoptosis. Moreover, PTCH1 engages in distinct interactions with ATG101 and ITM2A, effectively blocking the fusion of autophagosomes with lysosomes during the later stages of autophagy. This obstruction leads to the abnormal accumulation of autophagy markers and subsequently inhibits the progression of autophagy. Upper right: SHH non-canonical signaling pathway type II. SMO is activated by many stimuli, exerting its influence through dual mechanisms. First, upon receipt of the SHH signal, which activates SMO, TIAM1 is released, facilitating its translocation from the cytoplasm to the folds of the cell membrane. Once released and localized at the cell membrane, TIAM1 exerts its catalytic function by promoting GDP–GTP exchange on Rac1, thereby activating Rac1. Second, SMO activates AMPK, which promotes the transcription of GLI2, GLI3, HK2, PKM2, and LDHA while inhibiting GLI1 transcription. These events orchestrate the regulation of oligodendrocyte maturation and glycolysis and play a role in ferroptosis by upregulating the expression of xCT, GPX4, and GSH proteins. Lower: SHH non-canonical signaling pathway type III. Cross-talk between GLI and WNT, TGF-β, NOTCH, and KRAS signaling pathways. AKT: Protein kinase B; AMPK: AMP-activated protein kinase; AR: androgen receptor; ATG101: autophagy related 101; CARD: caspase recruitment domain; CKα1: casein kinase 1 alpha; CLIC4: chloride intracellular channel 4; CSL: CBF1, suppressor of hairless, Lag-1; ERK: extracellular signal-regulated kinase; GDP: guanosine diphosphate; GLI: glioma-associated oncogene; GPX4: glutathione peroxidase 4; GSH: glutathione; GSK-3β: glycogen synthase kinase 3β; GTP: guanosine triphosphate; Hepg2: human hepatocellular carcinoma cell line; mmc: mucosal mast cell; Hes1: hairy and enhancer of split 1; HK2: hexokinase 2; ITM2A: integral membrane protein 2A; LC3B: microtubule-associated proteins 1A/1B light chain 3B; LDHA: lactate dehydrogenase A chain; MAPK: mitogen-activated protein kinase; MEK: mitogen-activated protein kinase kinase; mTOR: mammalian target of rapamycin; NICD: Notch intracellular domain; p62: P62/SQSTM1, sequestosome 1; P63: p53 protein homolog; PI3K: phosphoinositol-3 kinase; PKM2: pyruvate kinase M2/M1; PTCH1: patched 1; RAC1: Ras-related C3 botulinum toxin substrate 1; RAS: rat sarcoma; RBPJ: recombination signal binding protein for immunoglobulin kappa J regions; SMO: smoothened; TCF/LCK: T-cell transcription factor/lymphoid-specific helix-loop-helix protein; TGF-β: transforming growth factor beta; TIAM1: T-lymphoma invasion and metastasis 1; ULK3: Unc-51 like autophagy activating kinase 3; WNT: Wingless-type MMTV integration site family; xCT: solute carrier family 7 member 11.

The SHH type I non-canonical pathway is characterized by the fact that PTCH1 does not interact with SMO but rather with other proteins. Studies have consistently demonstrated that the C-terminal domain of PTCH1 engages in functional interactions with autophagy-related 101, a pivotal autophagy-associated protein. Autophagy-related 101 plays a crucial role in initiating autophagy by facilitating autophagosome formation (Caballero-Ruiz et al., 2023). Furthermore, a previous study has revealed that PTCH1 may exert additional regulatory effects on autophagic flux through its interaction with integral membrane protein 2A, thereby expanding the complexity of its role in regulating autophagy (Morales-Alcala et al., 2021). PTCH1 is recruited by caspase-3, initiating a sequential activation cascade that leads to the activation of caspase-9, thereby triggering the apoptotic program (Kotulak-Chrząszcz et al., 2021). In addition, PTCH1 can interact selectively with cyclin B1, altering its typical subcellular distribution and leading to abnormal maturation-promoting factor phosphorylation. This aberrant phosphorylation ultimately causes the sequestration of maturation-promoting factor outside the nucleus, thereby inhibiting mitosis initiation (Barnes et al., 2001). These findings suggest the potential of the SHH signaling pathway to combat apoptosis and modulate cell proliferation (**Figure 3**).

#### Smoothed-mediated signaling

The SHH type II non-canonical pathway is SMO-dependent and involves GLI1-independent activity. SMO interacts with various proteins, particularly T-lymphoma invasion and metastasis 1 (TIAM1). The C-terminus of SMO interacts directly with TIAM1, maintaining TIAM1 in an inactive state during periods of inactivity. Upon activation of the SHH signal, SMO is activated, leading to the release of TIAM1, which translocates to the plasma membrane ruffles. There, TIAM1 activates Ras-related C3 botulinum toxin substrate 1 (Rac1) by catalyzing the guanosine diphosphate–guanosine triphosphate (GDP–GTP) exchange on Rac1, subsequently regulating actin remodeling and promoting the formation of dendritic spines. This process is independent of transcription (Sasaki et al., 2010). Another study showed that GSA-10 (a non-canonical SMO agonist) promotes GLI2 and GLI3 upregulation, downgrades GLI1 via SMO/AMP-activated protein kinase (AMPK) signaling, and efficiently increases the number of axonal contacts/ensheathments for each oligodendroglial cell (Del Giovane et al., 2022). SMO activation of AMPK can promote autophagy and lipid degradation (Akhshi and Trimble, 2021; Yao et al., 2023). Additionally, SMO regulates glycolysis in cells (Cui et al., 2024) and ferroptosis (Ma et al., 2023). These studies suggest that SMO is important in various cellular metabolic processes (**Figure 3**).

#### Glioma-associated oncogene-mediated signaling

The Type III non-canonical SHH signaling pathway involves intricate cross-talk with other signaling mechanisms, enabling the activation of GLI transcription factors independent of ligand binding. Numerous reports have documented the capacity of signaling pathways such as transforming growth factor-β (TGFβ), KRAS, Wnt/β-catenin, and NOTCH to activate GLI transcription factors (**Figure 3**).

*Wnt/*β*-catenin signaling pathway*

The Wnt signaling pathway predominantly encompasses the Wnt receptor Frizzled, LRP5/6 co-receptors, β-catenin, glycogen synthase kinase 3β, CK1 kinases, members of the T-cell factor/lymphoid enhancer-binding factor binding transcription factor family, and a suite of downstream target genes (Song et al., 2024). GLI3 contains T-cell factor/lymphoid enhancer-binding factor binding sites in the enhancers of the mouse telencephalon and has been confirmed as a direct target of Wnt/β-catenin signaling transduction (Paparidis et al., 2007). Furthermore, GLI2 can reduce the protein levels of Wnt/β-catenin pathway genes through small interfering RNA, such as downregulating the expression of leucine-rich repeat-containing G-protein coupled receptor 5 (Tanigawa et al., 2021). SHH/GLI and Wnt/β-catenin pathways also share similar regulatory molecules and pathways, including CK1ɑ (Han et al., 2019), glycogen synthase kinase 3β (Yang et al., 2022), and inflammatory pathways such as the SHH/Gli and Wnt/β-catenin pathways are jointly involved in activating the NLR family pyrin domain-containing 3 inflammasome (Zhou et al., 2024). These findings suggest that interactions between these two pathways are complex and bidirectional.

*Transforming growth factor-*β *signaling pathway*

The TGF-β signaling pathway is fundamentally structured by a suite of components, encompassing ligands, receptors, and an intricate network of signal transduction molecules. The Smad protein family plays a pivotal role in mediating downstream signaling events (Weiss and Attisano, 2013; Massagué and Sheppard, 2023). The TGF-β/Smad/GLI2 axis is considered essential for cancer metastasis. Liang et al. (2017) have identified TGF-β upregulated GLI2 in a Smad3-dependent manner and induced nuclear accumulation and DNA binding of GLI2. TGF-β1 also enhances the proliferation and invasion of the human hepatocellular carcinoma cell line by increasing the mRNA and protein levels of GLI1 (Sun and Wang, 2018). A study revealed that Smad3 and β-catenin protein in the WNT signaling pathway synergistically promote GLI2 transcription (Dennler et al., 2009). Activated GLI2 interacts with these two proteins to regulate chloride intracellular channel protein 4 (Wasson et al., 2022). Smad3 directly interacts with the Notch intracellular domain, thereby facilitating the activation of Notch downstream gene transcription (Klüppel and Wrana, 2005). Moreover, TGF-β induces Notch transcription via nuclear factor erythroid 2-related factor 2-dependent promoter activation (Yazaki et al., 2021).

*NOTCH signaling pathway*

The NOTCH signaling pathway comprises NOTCH receptor proteins, ligand proteins, Notch intracellular domain, recombination signal binding protein for immunoglobulin kappa J region, and accessory proteins (Guo et al., 2023). A previous study has found that Notch signaling controls the transcriptional or mRNA stability of all members of the GLI family in the spinal cord and that GLI may be a direct target of NOTCH signaling (Li et al., 2012). Marczenke et al. (2021) found that NOTCH signaling allows neural progenitor cells (NPCs) to respond to SHH signaling through GLI transcriptional regulation and the underlying GLI protein maintenance. The NOTCH pathway inhibits the transcription factor hes family bHLH transcription factor 1, which induces the activation of the SHH signaling pathway, leading to the activation of GLI1/2 (Lin et al., 2016). Additionally, C-promoter binding factor-1/recombination signal binding protein for immunoglobulin kappa J region is a transcriptional repressor that mediates NOTCH signaling, inhibits gene expression programs, lowers C-promoter binding factor-1, and upregulates the expression of unc-51-like kinase 3, which binds to and activates GLI2 (Goruppi et al., 2017). Another study discerned that NOTCH and TGF-β signaling pathways collaborate to orchestrate the expression of mucosal mast cell-specific protease genes, with Smad4 serving as a pivotal mediator in this regulatory process (Nakano et al., 2021).

*KRAS signaling pathway*

The KRAS signaling pathway is another pathway that exhibits cross-talk with and activates the SHH/GLI signaling cascade. KRAS primarily exerts its functions through the phosphoinositide 3-kinase (PI3K)/protein kinase B (AKT)/mammalian target of rapamycin (mTOR) and rapidly accelerated fibrosarcoma pathways (Luo, 2021), with the Rapidly Accelerated Fibrosarcoma/Mitogen-activated protein kinase (MEK)/mitogen-activated protein kinase axis directly regulating the activity of GLI1 (Po et al., 2017). Treatment with MEK inhibitors can reduce the phosphorylation of GLI1, while ERK can directly phosphorylate GLI1 (Ji et al., 2007). Furthermore, GLI transcription factors also mediate the promotion of p63 expression by the KRAS-Androgen Receptor axis (Wu et al., 2016).

## Effect of Sonic Hedgehog Signaling in the Central Nervous System

### Sonic Hedgehog signaling regulates the proliferation and differentiation of various neuronal cells

The canonical SHH signaling pathway plays an important role in the development of nerve cells. SHH-GLI signal transduction affects the fate of these cells, maintaining their undifferentiated state or promoting their proliferation, which is necessary in the early stages of neural stem cells (NSCs). SHH non-canonical signaling also appears to be involved, with the SHH and NOTCH pathways working together to promote NSC proliferation during neocortical development (Ohtsuka and Kageyama, 2022). Notably, SHH signaling plays a pivotal role in maintaining the physiological proliferation of granular neuron progenitors (GNPs) during cerebellar development (Hu et al., 2024). The SHH signaling upregulates β-catenin, leading to the maintenance of a higher proportion of cerebellar granular neuron progenitors (CGNPs) in a proliferative state (Mani et al., 2020). In addition, SHH signaling is essential for differentiating gamma-aminobutyric acid (GABA)-ergic neuronal precursors in the nucleus of the medial longitudinal fasciculus (Wang et al., 2023b). *In vitro*, SHH modulates the proliferation and differentiation of NPCs under inflammatory stress (Tail et al., 2022). Activation of SHH-SMO maintains oligodendrocyte precursors in an undifferentiated state (Nocera et al., 2024). Additionally, PTCH1 interacts with G protein-coupled receptor 37 like 1, facilitating its internalization and trafficking of PTCH1 itself. This process subsequently triggers the activation of the SHH pathway, ultimately leading to the upregulation of proliferative signaling in astrocytes (La Sala et al., 2020). This intricate network of SHH signaling, involving diverse neuronal cell interactions, underscores its functionally extensive and multifaceted nature within the CNS.

### Sonic Hedgehog signaling regulates neurogenesis

Neurogenesis persists in most mammals, including the human hippocampus, and is most active during the embryonic stage in animals (Eriksson et al., 1998). In mammals, the subgranular zone and subventricular zone (SVZ) of the lateral ventricles retain the ability of NSCs to proliferate and generate new neurons (Niklison-Chirou et al., 2020). Previous studies have shown that the SVZ produces various types of interneurons (Huang et al., 2022a) and that the subgranular zone is involved in producing excitatory neurons critical for memory development (Angelopoulos et al., 2022). The importance of SHH signaling in regulating neurogenesis within the SVZ is demonstrated by the observation that SMO-knockout mice exhibit markedly reduced Ki67^+^ and DCX^+^ cells in the SVZ, leading to decreased neurogenesis in the SVZ and subgranular zone of young adult mice. Moreover, these mice displayed an accelerated depletion of neurogenic cells during the aging process (Wang et al., 2022a). In mouse embryos with SHH gene knockout, Nkx2.1^+^ cells were decreased in the medial ganglionic eminence, resulting in fewer cortical interneurons (Cheng et al., 2023). O-GlcNAc transferase regulates GNP neurogenesis by binding to the S355 site of GLI2 within the SHH signaling pathway, inhibiting its acetylation and preserving its transcriptional activity (Chen et al., 2022). Recent studies have found that pro-opiomelanocortin neurogenesis is regulated by SOX9^+^ hypothalamic ventricular zone (VZ) progenitors, with SHH essential for the development and survival of SOX9^+^ progenitors. The NOTCH pathway supports SHH signaling within the hypothalamic VZ (Place et al., 2022). Furthermore, SHH modulates the expression of SOX2 via Transient Receptor Potential Canonical 3-mediated Ca^2+^ signaling, suppressing the propagation of canonical signals during this process. Consequently, SHH shifts its function by promoting cell proliferation to facilitate neuronal differentiation, ultimately affecting neurogenesis by disrupting the balance between the two processes (Shim et al., 2023). This further emphasizes the importance of SHH signaling in maintaining and modulating neurogenesis.

Recent advances in stem cell therapy have highlighted the critical role SHH signaling in promoting neuroregeneration and neural repair (Dwivedi et al., 2025). When combined with retinoic acid, SHH stimulation promotes the conversion of dental pulp stem cells into working motor neurons, substantially improving their capacity to restore neural function (Darvishi et al., 2021). Moreover, activation of the SHH-Gli signaling axis facilitates the differentiation of human induced pluripotent stem cells into dopaminergic neural precursor cells by regulating key neuronal fate determinants, including Nestin and Sox2, which are essential for neuronal lineage specification (Lyu et al., 2023). Additionally, SHH significantly enhances the proliferation of menstrual blood-derived stem cells and induces their differentiation into neuron-like cells. SHH-activated menstrual blood–derived stem cells further contribute to neural repair via secretion of anti-inflammatory factors, which alleviate inflammatory responses and promote functional recovery (Shi et al., 2023b). Collectively, these findings highlight the multifaceted role of SHH in stem cell–based therapeutic strategies for neural regeneration and repair.

### Sonic Hedgehog signaling regulates axon

The SHH pathway critically influences central nervous system development through its regulation of neuronal process extension and directional growth (González-Castrillón et al., 2023). Axons are guided by guidance molecules located along specific pathways. A recent study has shown that defects in GLI2 disrupt the SHH signaling pathway, influencing the developmental trajectory of hypothalamic cortical axons and ultimately leading to misguided projection towards inappropriate regions (Callejas-Marin et al., 2022). The SHH-PTCH1-GLI1 signaling axis also plays an axonal guiding role (Xiao et al., 2021). Corticospinal-specific SHH overexpression promotes axonal sprouting (Wu et al., 2024). Experimental evidence reveals that SHH-treated neurons exhibit approximately twofold increases in axonal elongation relative to untreated controls (Stubbs et al., 2022). Further research has found that SHH stimulation in dendrites accelerates axon outgrowth (Yin et al., 2020), enabling reaching its presumptive postsynaptic target cell more quickly. In addition, SMO influences the cytoskeleton assembly of axonal growth cones by activating Src Family Kinases (SFK), specifically through β-arrestins serving as scaffold proteins to bridge SMO and SFK during SHH-mediated axonal guidance (Sauvé et al., 2024), thereby activating SFK by non-canonical signaling and directing axonal growth towards increasing concentrations of SHH (Yam et al., 2009). Considering these multifaceted roles in neuronal process development, SHH likely contributes substantially to optimizing neural communication efficiency within the CNS’s intricate network.

### Sonic Hedgehog signaling regulates the neurotransmitters

Neurotransmitters (NTs) are crucial chemical messengers throughout the CNS and peripheral nervous system, enabling signal amplification, transduction, and conversion that support fundamental cognitive processes and behavioural regulation. The SHH pathway has significant influence over NTs through modulation of receptor and transporter expression, directly affecting NT production and release mechanisms (Banerjee et al., 2020). Notably, SHH signaling activation modulates the expression of various NT receptors and transporters, thereby influencing the synthesis and release of NTs (Prajapati et al., 2023). A recent study has shown that SHH non-canonical SMO signaling promotes human serotonin expression (Gupta et al., 2022). SHH signaling exerts regulatory influences on various NT systems (Vazin et al., 2014), notably the cholinergic system, which is distinctively marked by its reliance on the NT acetylcholine. It is important for the development and emergence of cholinergic neurons within the basal forebrain (Muñoz et al., 2020). Moreover, it regulates the expression of dopaminergic receptors (Qiu et al., 2019) involved in reward and motor control, modulating dopamine-mediated behaviors. Exogenous activation of SHH signaling in the spinal cord rapidly increases brain-derived neurotrophic factor expression (Delmotte et al., 2020). SHH has also been shown to increase brain-derived neurotrophic factor levels in primary cortical neurons (He et al., 2016). Additionally, SHH non-canonical signaling regulates glutamate receptor expression by inducing excitatory amino acid transporter 2a expression (Hentig et al., 2021a). These multifaceted interactions position SHH as a key modulator of neural protection and metabolic support beyond its traditional signaling functions.

### Sonic Hedgehog signaling regulates the neuroinflammation

Neuroinflammation, as a defense mechanism, responds to various external stimuli (Sanjay et al., 2022; Zhang et al., 2024). However, its excessive activation has emerged as a critical factor in the progression of numerous CNS diseases (Cho et al., 2024). Characterized by resident glial cell activation, pro-inflammatory cytokines release, blood–brain barrier (BBB) disruption, and neuroinflammation, significantly impact CNS homeostasis (Zhou et al., 2024; Cantone et al., 2025). The SHH signaling pathway has been identified as a potential modulator of neuroinflammation by influencing these processes.

Silent information regulator factor 2–related enzyme 1 stimulates Gli1 nuclear translocation to initiate downstream signaling cascades, inhibiting the activation of microglia and inflammation (Liao et al., 2023). Furthermore, the inhibition of the SHH signaling pathway may lead to abnormal activation of microglia in the hippocampus of mice (Tan et al., 2024). Additionally, abnormal peripheral lipid metabolism causes inflammatory mediators to enter the brain by increasing the permeability of BBB, which activates microglia to release more inflammatory factors, thereby triggering neuroinflammation (Zingale et al., 2022). SHH may participate in this process by inducing lipid metabolic disorders through reducing insulin resistance (Garg et al., 2022). In addition, PTCH1 is activated by miRNA-9‐5p, which reduces inflammatory cytokine release and decreases neuroinflammation (Wu et al., 2020a). Interestingly, neuroinflammation can also affect the epigenetic transcriptional control of SHH signaling pathway members. Recent studies have shown that neuroinflammation alters the DNA methylation status of the promoter regions of SHH signaling pathway members by regulating the gene expression of DNA methyltransferases (Costa et al., 2023). Specifically, after neuroinflammation induction, the promoters of *SHH*, *PTCH1*, and *SUFU* genes exhibit DNA hypermethylation in the hippocampus, while in the striatum, the promoter of the *SUFU* gene shows DNA hypomethylation (Costa et al., 2023).

### Sonic Hedgehog signaling regulates neuronal and astrocyte mitochondria

Mitochondria play an important metabolic role as energy providers in nerve cells (Adam et al., 2025; Izquierdo, 2025). A previous study reported that the activation of SHH-GLI1 signaling reduces mitochondrial fission and promotes mitochondrial elongation by inhibiting dynamin-related protein 1 (Yao et al., 2017). Additionally, smoothened agonist (SAG), a non-canonical SHH agonist, restores mitochondrial quality and the balance between fission and fusion in abnormal astrocytes following SAG treatment (Vicente-Acosta et al., 2022). SAG balances the expression between mitofusin 2 and dynamin-related protein 1 in the mouse prefrontal cortex, significantly reversing the phenomena of mitochondrial fragmentation, shrinkage, and declines in size parameters such as area, perimeter, and circularity, ultimately exerting neuroprotective effects (Sun et al., 2023). Neurons exhibit mitochondria characterized by heightened electron density, enhanced respiratory activity, and a stabilized membrane potential upon treatment with SHH (Yao et al., 2017). This is accompanied by increased levels of mitochondrial DNA, peroxisome proliferator-activated receptor gamma coactivator 1-α, and mitochondrial transcription factor A (Jia et al., 2023). Mitochondrial membrane potential, a key indicator of mitochondrial function (Perry et al., 2011), is preserved in cortical neurons treated with SHH, indicating that SHH protects mitochondrial function (He et al., 2017). In summary, SHH regulates the structure and function of mitochondria, thereby influencing the cellular life cycle in the CNS (**Additional Table 1**).

**Additional Table 1:** SHH regulates several aspects of the central nervous system

Functions	Functional segments	Mechanisms	References
Regulates the proliferation and differentiation of nerve cells	Neural stem cells	SHH signaling pathway activation	Ohtsuka and Kageyama, 2022
	GABAergic neuronal precursors	Conditionally blocking Shh signaling inhibits the proliferation of GABAergic progenitor cells and reduces the number of Pax^2+^ cells	Wang et al., 2023b
	Oligodendrocyte precursors	Activation of SMO in the SHH pathway promotes the proliferation of OPCs but inhibits their differentiation	Nocera et al., 2024
	Astrocytes	PTCH1 interacts with GPR37L1, facilitating its internalization and trafficking, and subsequently triggers activation of the SHH pathway.	La Sala et al., 2020
	Granular neuron progenitors	By upregulating the N-myc to increase the levels of the protein 3-Catenin, thereby promoting the proliferation of CGNPs	Mani et al., 2020
Regulates neurogenesis	SVZ and subgranular zone regions	The reduction of SMO expression leads to decreased migration of immature neurons	Wang et al., 2022a
	Amygdalar region	Overexpression of the SHH gene increases neurogenesis	Hung et al., 2015
	Arcuate nucleus	SHH and Notch regulate SOX9^+^ progenitors to govern arcuate POMC neurogenesis	Place et al., 2022
Regulates axon	Axonal guidance	Bridging SMO and SFK during SHH-mediated axon guidance via 3-arrestin as a scaffold protein, activates SFK and directs axonal outgrowth	Sauvé et al., 2024
	Axonal growth	Knockdown of SUFU activates the SHH signaling pathway, which enhances cell survival and promotes massive axon growth.	Chen et al., 2024c
Regulates the secretion of neurotransmitters	Serotonin	Activation of the SMO-SHH signaling pathway	Gupta et al., 2022
	Acetylcholine	The SHH signaling pathway promotes the survival and function of cholinergic neurons and regulates the secretion of acetylcholine	Muñoz et al., 2020
	Dopamine	Elevating the levels of SHH expression augments the expression of Nurrl, consequently leading to an upregulation of Tyrosine Hydroxylase protein levels.	Wu et al., 2024
	BDNF	SAG activates SMO to promote BDNF expression through no-canonical SHH signaling pathways	Delmotte et al., 2020
	Glutamate	Prophylactic activation of SHH upregulates the concentration of Eaat2 to reduce extracellular glutamate	Hentig et al., 2021a
Regulates neuronal mitochondria	Mitochondrial fission/fusion	SHH signaling activity reduces mitochondrial fission and promotes mitochondrial elongation by inhibiting the DRP1 protein	Vicente-Acosta et al., 2022
	Mitochondrial membrane potential	CMXRos of neurons were significantly increased after SHH treatment	Yao et al., 2017
	Microglia	Sirt1 promotes the nuclear translocation of Glil and activates its downstream signaling cascade, thereby inhibiting microglial activation and inflammation.	Liao et al., 2023
	Inflammatory factors	Activation of the SHH signaling pathway inhibits the expression of IL-6 and TNF-α	Wu et al., 2020b

BDNF: Brain-derived neurotrophic factor; CGNPs: cerebellar granule neuron progenitors; CMxRos: Cre-Mertk-Rosa26; Drp1: dynamin-related protein 1; Eatt2: excitatory amino acid transporters 2; GABA: gamma-aminobutyric acid; GPR3TL1: G protein-coupled receptor 3, transmembrane domain and thyrotrophin-releasing hormone gene-like 1; IL-6: interleukin-6; N-myc: neuroblastoma Myc oncogene; Nurr1: nuclear receptor related 1; OPCs: oligodendrocyte progenitor cells; POMC: pro-opiomelanocortin; PTCH1: patched 1; SAG: Sonic Hedgehog activated gene; SFK: Src family kinase; SHH: Sonic Hedgehog; Sirt1: silent information regulator factor 2-related enzyme 1; SMO: smoothened; SUFU: suppressor of fused; SVZ: subventricular zone; TNF-α: tumor necrosis factor-α.

## Sonic Hedgehog Signaling Pathway in Central Nervous System Injuries

### Sonic Hedgehog signaling pathway in depression

The pathogenesis of depression encompasses various theories, including NT-receptor and neuroplasticity hypotheses. However, these frameworks fail to comprehensively elucidate the underlying pathological mechanisms because of the complex nature of depression. Recent years have seen increased focus on the intricate interplay between neuronal function, neurogenesis, and developing depressive disorders as promising avenues for further understanding and potential therapeutic interventions.

**Figure 4:**
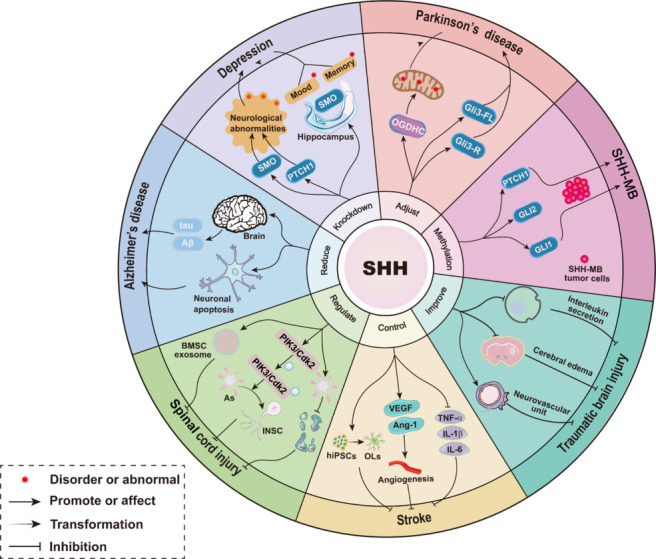
Role of SHH in central nervous system disorders. SHH modulates many events among disorders, including depression, Parkinson’s disease, traumatic brain injury, spinal cord injury, Alzheimer’s disease, stroke, and SHH-MB. Ang-1: Angiopoietin-1; AS: astrocyte; Aβ: amyloid-β; BMSC: bone marrow stromal cells; cdk2: Cyclin-dependent kinase 2; GLI: glioma-associated oncogene; hIPSCs: human induced pluripotent stem cells; IL-1β: interleukin-1β; IL-6: interleukin-6; INSC: islet neuroendocrine stem cells; OGDHC: oxoglutarate dehydrogenase complex; OLs: oligodendrocytes; PIK3: phosphatidylinositol 3-kinase; PTCH1: Patched 1; SHH: Sonic Hedgehog; SHH-MB: Sonic Hedgehog-medulloblastoma; SMO: smoothened; TNF-α: tumor necrosis factor-α; VEGF: vascular endothelial growth factor.

Abnormalities in hippocampal neurogenesis contribute significantly to depression. Abnormal oxidative stress and neurogenesis have been observed in the hippocampi of chronic unpredictable mild stress rats (Fatima et al., 2020). The reduction of PTCH1 and SMO protein levels has been associated with hippocampal neurogenesis abnormalities (Antonelli et al., 2018; Wang et al., 2022a), while the upregulation of SHH mRNA expression and the subsequent activation of signaling pathways fosters neurogenesis and ameliorates depressive-like behaviors (Fatima et al., 2019). The chronic unpredictable mild stress rats showed marked downregulation in the expression of SHH signaling markers within the dorsal and ventral hippocampus, it is especially pronounced in the ventral hippocampus (Tayyab et al., 2018). Notably, SHH ablation, specifically in the dentate gyrus of the dorsal hippocampus, results in memory deficits by suppressing the experience-dependent activation of immature neurons. Moreover, SHH knockout in the dentate gyrus of the ventral hippocampus elicits emotional disorders by impeding the maturation of immature neurons. This differential response highlights the regional specificity and critical role of SHH signaling in modulating cognitive and emotional functions within distinct hippocampal subregions (Luo et al., 2024; **Figure 4**).

### Sonic Hedgehog signaling pathway in Parkinson’s disease

Parkinson’s disease (PD) is a progressive neurodegenerative condition characterized by tremors and bradykinesia (Yu et al., 2024). It involves the gradual deterioration of the nigrostriatal dopaminergic pathway, accompanied by dopamine depletion (Eser et al., 2024). Mitochondrial dysfunction is increasingly recognized as a pivotal contributing factor to the pathogenesis of PD (Jankovic and Tan, 2020). Disturbances in NTs, particularly gamma-aminobutyric acid, further contribute to disease progression (O’Gorman Tuura et al., 2018).

Dopaminergic neurons play crucial roles in PD. Research has revealed a novel mechanism involving the non-canonical SHH pathway, wherein the Stil protein alleviates SUFU-mediated suppression of GLI transcription factors and promotes SHH-mediated gene expression that supports dopaminergic neuron survival and renewal (Li et al., 2019). When astrocytes were induced to differentiate into dopaminergic neuron-like cells, the SHH supplementation significantly improved the conversion rates (Chen et al., 2024b). Similarly, mesenchymal stem cells (MSCs) differentiation into dopaminergic phenotypes appears SHH-dependent (Wang et al., 2021). Growing evidence suggests decreased levels of GLI3-FL and GLI3-R proteins in the dopaminergic neurons of patients with PD, indicating impaired GLI3 processing and overactivation of SHH signaling (Schmidt et al., 2022; **Figure 4**). In contrast, abnormal SHH signaling reduces the activity of succinate dehydrogenase, an enzyme in mitochondria (Schmidt et al., 2023). These molecular disturbances lead to structural abnormalities in cellular cilia and impair energy production through oxidative phosphorylation, both of which represent important pathological features in PD development (Imaizumi et al., 2015). Therefore, modulating SHH signaling may help restore normal cellular metabolism, slowing or preventing PD progression.

### Sonic Hedgehog signaling pathway in Alzheimer’s disease

Alzheimer’s disease (AD) is a common neurodegenerative disorder that primarily affects memory, thinking, and behavior (De Strooper and Karran, 2016). The pathological features of AD mainly include two aspects: the deposition of extracellular senile plaques and the formation of intracellular neurofibrillary tangles (Wang et al., 2023c). Senile plaques are composed of amyloid-β (Aβ) deposits, whereas neurofibrillary tangles are composed of hyperphosphorylated tau protein (Du et al., 2021).

SHH is involved in various stages of AD and affects multiple pathological processes (Prajapati et al., 2023). Neuron cells with *SHH* gene knock exhibited a stronger tendency towards apoptosis (Li et al., 2021a). Further research indicates that SHH mediates Aβ-dependent cell cycle re-entry, resulting in apoptosis of nerve cells (Chao et al., 2019). This suggests that SHH affects the pathogenesis of AD by regulating the expression of Aβ and tau proteins and neuronal apoptosis (**Figure 4**). Interestingly, mature neurons typically maintain SHH signaling in an inactive state during normal physiological conditions. However, exposure to cellular stressors including oxygen deprivation or ischemic injury triggers substantial upregulation of SHH expression (Chung et al., 2022). This differential response implies SHH may serve protective functions in neurons facing adverse environmental challenges.

### Sonic Hedgehog signaling pathway in spinal cord injury

Spinal cord injury (SCI) is a catastrophic injury to the CNS that causes temporary or permanent changes in its function (Feng et al., 2023). The pathophysiology of SCI includes primary and secondary injury (Tian et al., 2023). Primary injury is mechanically induced injury to the spinal cord tissue, and secondary injury is an overreaction to SCI after a primary injury, which involves the immune, nervous, and vascular systems, including apoptosis, inflammation, ischemia, oxidative stress, and demyelination (Hu et al., 2023). Axonal and cell membrane disruption and inflammation dominate the initial post-injury phase, followed by microglial and astrocyte proliferation and activation (Hutson and Di Giovanni, 2019).

Recently, NSC therapies have surfaced as a promising avenue for addressing neural injuries and neurodegeneration, assuming a pivotal role in strategies aimed at repairing SCI. These crucial interneurons can originate from primitive interneurons residing within the spinal cord or may be differentiated from transplanted stem cells and endogenous NSCs, marking an advancement in the field (Yu et al., 2023). Mature astrocytes can be directly converted into induced neural cells via Octamer-binding transcription factor 4 (Oct4) (Díaz, 2019). SHH can enhance the efficiency of Oct4-driven reprogramming of astrocytes into induced NSCs (Yang et al., 2019; **Figure 4**). Further studies suggested that Oct4 and SHH cooperate through Sox2/SHH-mediated downstream signaling and cross-talk with the PI3K/Cyclin-dependent kinase 2/Smad ubiquitination regulatory factor 2 signaling pathway to initiate astrocyte reprogramming into NSC (Keenan et al., 2001). This is because SHH treatment increased the expression of phospho-PI3K and Cdk2. New evidence suggests that the activation of SHH signaling is necessary for MSC-derived exosome-mediated SCI repair (Tail et al., 2022). Another study found that exosomes from bone MSC improved SCI symptoms in SCI model rats, accompanied by increased GAP-43 expression and decreased apoptotic cells (Jia et al., 2021). In addition, the transplantation of SHH into SCI rats with bone MSCs significantly improved SCI function and promoted neurogenesis (Gu et al., 2022). The effects of exosomes on SCI were ineffective in SHH-silenced rats. Furthermore, SHH exerts anti-inflammatory effects on SCI (Zhang et al., 2021). In contrast, a study examining the role of the SHH-GLI1 signaling pathway in blood–spinal cord barrier permeability following SCI demonstrated that activation of this pathway may quell inflammatory responses and apoptosis, subsequently reducing blood–spinal cord barrier permeability (Yue et al., 2020). Specifically, the SHH-GLI1 signaling pathway regulates the behaviour and function of endothelial cells and astrocytes intimately associated with the blood–spinal cord barrier (Zhao et al., 2022).

### Sonic Hedgehog signaling pathway in traumatic brain injury

Traumatic brain injury (TBI) is caused by a bump, blow, or jolt to the head or a penetrating head injury that disrupts normal brain function (Timofeev et al., 2012). TBI entails two phases of injury: an initial head trauma via an external force that results in mechanical damage to brain tissue and a secondary biochemical cascade (Wang et al., 2022b). Primary injury, including a direct hit, can produce indirect damage through the acceleration-deceleration mechanism, penetrating versus non-penetrating, and secondary injury, including ischemia, causes cell death, cytogenic edema, apoptosis, mitochondrial dysfunction, cortical diffusion inhibition, and axonal damage (Khellaf et al., 2019). Research suggests that SHH signaling provides a basis for the migration of cerebellar crest cells to the cerebellar granule cell layer and their differentiation into neurons, thus ameliorating TBI for neurogenesis and other neural responses (Hentig et al., 2021b). In addition, exogenous SHH alleviates cerebral edema and neuronal apoptosis and promotes the recovery of neural function in rats after TBI (Wu et al., 2020b; **Figure 4**). A previous study has shown that the activation of SHH signaling can stabilize the neurovascular unit, thereby reducing damage to tight junction structures (Xia et al., 2013) and inhibiting the release of inflammatory cytokines and neuronal apoptosis. Research has discovered that the localized restoration of SHH in rodent brains can maintain endogenous cell proliferation and ameliorate TBI-induced motor deficits (Pringle et al., 2021).

### Sonic Hedgehog signaling pathway in Sonic Hedgehog medulloblastoma

Brain tumors encompass a diverse group of neoplasms, including glioblastoma multiforme (GBM), MB, and other types of brain tumors (Tang-Schomer et al., 2022). SHH signaling pathway plays a pivotal role in the initiation and progression of brain tumors by promoting tumor cell proliferation (Longhitano et al., 2023), maintaining cancer stem cell properties (Okonechnikov et al., 2023), facilitating tumor invasion and metastasis (Chen et al., 2024a), and inducing angiogenesis (Agrawal et al., 2023). Notably, activation of the SHH pathway is frequently observed in GBM, with high levels of GLI1 and GLI2 being significantly associated with poor patient prognosis (Shi et al., 2023a). Moreover, aberrant activation of the SHH pathway has been implicated in the development of drug resistance in GBM, such as temozolomide resistance, which is associated with elevated GLI1 expression (Lobón-Iglesias et al., 2023).

Sonic Hedgehog-medulloblastoma (SHH-MB), a subtype of medulloblastoma, originates from CGNPs with active SHH signaling, accounting for approximately one-third of all medulloblastomas (Schüller et al., 2008). In addition to SHH-MB, there are three other subtypes: WNT, Group 3, and Group 4 (Taylor et al., 2012). SHH MB primarily occurs in the cerebellar hemispheres but can also be found in the vermis of the cerebellum. It is characterized by abnormal activation of the SHH pathway, which may be due to mutations in the *PTCH1*, *SUFU*, and *SMO* genes (Lewis et al., 2004; Jones et al., 2012), as well as Tumour protein 53 mutations and GLI2 amplification (Kool et al., 2014). Recent discoveries have indicated that methylation of key components in the canonical SHH pathway is another major factor. Protein arginine methyltransferase 5, through direct methylation of GLI1, enhances the protein stability of GLI1, thereby modulating the activity of the signaling pathway and impacting the progression of SHH-MB (Wynn et al., 2022). Furthermore, methyltransferase-like 3 augments the RNA stability and translation efficiency of PTCH1 and GLI2 through RNA N6-methyladenosine methylation, which in turn promotes the transcriptional activity of GLI2, driving the growth and migration of SHH-MB tumor cells (Zhang et al., 2022; **Figure 4**). When PTCH1 undergoes loss-of-function mutations, it fails to effectively inhibit the transmission of SHH signals by SMO. This regulatory failure permits continuous pathway activation, driving excessive multiplication of granulosa cell progenitors, genomic instability, and alterations in the tumor microenvironment, creating conditions favorable for SHH-medulloblastoma formation (van Essen et al., 2024). Furthermore, atonal bHLH transcription factor 1 (Atoh1) maintains PC in GNPs by transcriptionally regulating centrosomal protein 131, a centrosome satellite protein (Chang et al., 2019), while enables GNPs to respond effectively to SHH signaling. A study points out a positive feedback relationship between Atoh1 and the SHH signaling pathway (Chang et al., 2019), and further accelerates the development of SHH-MB. The role of non-coding RNAs in SHH-MB is increasingly emerging. Researches have revealed that the knockdown of Hedgehog interacting protein-anti-sense 1 causes disruptions in the mitotic spindle structure of SHH-MB cells, increases DNA damage, and consequently restricts the proliferation of tumor cells (Bartl et al., 2022). Another regulatory element, circular RNA circ-63706, suppresses the proliferation, migration, and invasion of cells when depleted in SHH-MB models. This non-coding RNA participates in the regulation of lipid homeostasis and maintenance of redox balance, influencing the onset of SHH-MB (Katsushima et al., 2023).

### Sonic Hedgehog signaling pathway in stroke

Stroke, a prevalent neurological disorder, is characterized by insufficient cerebral blood supply or loss of vascular integrity, mainly classified as ischemic and hemorrhagic (Shehjar et al., 2023). Ischemic strokes account for about 80% of all stroke cases, which are often triggered by vascular pathologies such as atherosclerotic plaque formation and endothelial dysfunction (Jiang et al., 2013). Conversely, hemorrhagic strokes account for 20% of cases (Xu et al., 2025), which is mainly due to cerebral vascular rupture, leading to intracerebral or subarachnoid hemorrhage (Zhang et al., 2023).

The SHH signaling pathway is involved in stroke development, particularly in regulating glial cell activity. Under ischemic conditions, the activity of the SHH signaling pathway is inhibited by the degradation of Scube2, which reduces the expression of tight junction protein and disrupts the interaction between astrocytes and endothelial cells, resulting in BBB damage (Shi et al., 2024). Fibrous scar formation is a typical pathological feature of ischemic stroke (Wang et al., 2022c), and the SHH signaling pathway affects this process by regulating the M2-type polarization of microglia (Yang et al., 2024). TGFβ1 and platelet-derived growth factor subunit a secreted by M2 microglia are key factors in fibroblast activation, and the activation of SHH signaling pathway can promote the expression of these factors, further contributing to fibrous scar formation.

Stem cell therapy has become a research hotspot in cell replacement therapy for ischemic stroke (Yamaguchi et al., 2022). Xu et al. (2022) established a stable chemically defined protocol to generate a large number of transplantable and functioning oligodendrocytes from human induced pluripotent stem cells by partial inhibition of SHH activity and sequential induction of oligodendrocyte transcription factor 2 overexpression. In the rat model of transient middle cerebral artery occlusion, the transplantation effectively promoted neurological recovery by reducing neuronal death, promoting remyelination, and rescuing spatial memory decline. In addition, the SHH signaling pathway also plays an important role in angiogenesis (Wu et al., 2024). Activation of SHH signaling can promote the expression of angiogenesis factors vascular endothelial growth factor and angiogenin, enhance the migration ability of endothelial cells, and thus promote angiogenesis (Hui et al., 2022). Interestingly, SHH can not only promote angiogenesis through the cell’s autocrine expression, but also act on endothelial cells through paracrine mechanisms, further promoting angiogenesis (Dai et al., 2023). Furthermore, activation of the SHH reduces the expression of pro-inflammatory cytokines, such as interleukin-1β and interleukin-6, which play a key role in the inflammatory response following a stroke (Ghasemi et al., 2022; **Figure 4**).

## Sonic Hedgehog as Potential Therapeutic Targets

Over the past two decades, significant efforts have been directed towards elucidating the intricate interplay between the SHH signaling pathway and an array of pathological conditions underlying CNS disorders. The focus on the molecular and proteomic landscapes offers immense potential for nuanced manipulation to foster equilibrium in disease states and enhance the efficacy of pharmacological interventions. Integrating existing therapeutic modalities has highlighted the substantial value of SHH-signaling agonists in disease prevention and treatment strategies. Concurrently, there has been a paradigm shift in research, with investigators diverting attention from the canonical SHH pathway to the non-canonical pathway. Consequently, a repertoire of drugs targeting these pathways has been developed, broadening the strategies available for the clinical management of CNS diseases (**Additional Table 2**).

**Additional Table 2:** Drugs for the treatment of CNS disease by targeting SHH signaling

Drug	Target	Disease	Object	Potential effect	Reference
Gami-Shinkiwhanin	Promote the expression of SHH protein	Depression	Aging mice	Antidepressant effects	Park et al., 2018
Ropinirole	Promote the expression of SHH protein	Depression	CUMS Wistar rats	Increase neurogenesis in the hippocampus	Fatima et al., 2020
Naringenin	Up-regulate expression of SHH and GLI	Depression	CUMS Wistar rats	Mitigate morphological anomalies in the hippocampal; antidepressant effects	Tayyab et al., 2019a
Nicotine	Increase SHH signaling cascade	Depression	CUMS Wistar rats	Improve cognitive impairment; antidepressant effects	Tayyab et al., 2019b
Neogenin	Induce SHH signaling to GlI1	Depression	HepG2 cells	Regulate adult hippocampal neurogenesis	Sun and Wang, 2018
Leonurine	Activate the SHH/GLI signaling pathway	Depression	CUMS SD rats	Restore gut microbial metabolic homeostasis	Meng et al., 2023
*Zuogui Jiangtang Jieyu* Decoction	Activate SHH signaling	Depression	Diabetes mellitus complicated by depression SD rats	Improve the self-renewal ability of neural stem cells; antidepressant effects	Yang et al., 2023
MiR-210-5p	Bind to SUFU to activate SHH signaling	Parkinson's disease	hiPSC lines	Promote hiPSCs differentiation into midbrain dopaminergic precursors.	Lyu et al., 2023
MiR-124	Activate the SHH signaling pathway	Parkinson's disease	PD model mice	Promote dopamine receptor expression and neuronal proliferation and suppress neuronal apoptosis	Wang et al., 2019b
MiR-7	Activate the SHH signaling pathway	Parkinson's disease	Zebrafish	Promote the development and differentiation of dopaminergic neurons	Adusumilli et al., 2020
Astragaloside IV	Upregulate of SHH signaling	Parkinson's disease	NSCs from mesencephalons of SD rat embryos	Increase NSCs proliferation and differentiation	Gao et al., 2018
*Zhichan* Decoction	Promote the expression of SHH mRNA	Parkinson's disease	NSCs of PD model rats	Promote neural stem cell differentiation	Wentao et al., 2011
Purmorphamine	Activate SHH signaling pathway	Parkinson's disease	PD model mice	Anti-inflammatory and neuroprotective effects	Shao et al., 2017
Epigallocatechin-3-gallate	Activate SHH signaling pathway	Parkinson's disease	Adult hippocampal NPCs from mice	Neuroprotective and neuroregenerative effects	Wang et al., 2012
*Lycium barbarum* polysaccharide	Activate of the SHH signaling pathway	Alzheimer's disease	Mouse Neuro-2a cells	Neuroprotective effects	Zhao et al., 2016
Resveratrol	Activate SHH signaling	Spinal cord injury	NIH3T3 cells	Enhance NIH3T3 cell viability	Guo et al., 2018
MiR-153	Activate the SHH signaling pathway.	Traumatic brain injury	MCAO rats	Promote angiogenesis	Wang et al., 2019a
BQ788	Up-regulate expression of SHH protein	Traumatic brain injury	TBI model mice	Reduce the permeability of the blood-brain barrier	Michinaga et al., 2021
Trametinib	Inhibit MEK1/2, thereby reducing the expression of SHH pathway genes	SHH-MB	Human and mouse SHH-MB tumor cells	Reduce tumorsphere size, stem/progenitor cell proliferation, viability, and migration	Borlase et al., 2023
DS-01-38	Inhibit SHH signaling and reduce the expression of GLI1	SHH-MB	MB21 cell from medulloblastoma tumors of Ptch^+/-^mice	Inhibit cell proliferation, activate DNA damage and apoptosis responses.	Hwang et al., 2024
Arsenic trioxide	Reduce the expression or activity of GLI protein	SHH-MB	Pediatric SHH-MB cell lines	Increased oxidative stress promotes apoptosis in tumor cells and blocks the cell cycle	Dos Santos Klinger et al., 2020
Vismodegib	Disrupt the HH pathway through SMO antagonism	SHH-MB	Child	Inhibit tumor cell proliferation	Robinson et al., 2017
HUWE	Promote the ubiquitination of TTBK2 and subsequent degradation to regulate the SHH signaling pathway	SHH-MB	TTBK2 knockout Daoy cells	Regulating primary cilia promotes GNP differentiation	Lin et al., 2024
Taurine	By inhibiting the expression of PTCH1, the SHH signaling pathway is activated	Stroke	Focal cerebral cortical ischemia mice model	Promote axonal budding and improves mitochondrial function	Jia et al., 2023
Guanxinning	Up-regulated the expression of SHH and promoted the binding of SHH to PTCH1	Stroke	Cerebral ischemia-reperfusion injury	Reduce the volume of cerebral infarction, and cerebral edema, and reverse the permeability of the blood-brain barrier.	Xiao et al., 2021

CUMS: Cerebral microvascular endothelial cells; DS-01-38: (2-Ethyl-5-(1-methyl-1H-pyrazol-4-yl) benzofuran-3-yl) (4-hydroxyphenyl) methanone; GLI: glial cells; HepG2: human hepatocellular carcinoma; HIPSC: human induced pluripotent stem cells; MCAO: middle cerebral artery occlusion; MEK1/2: mitogen-activated protein kinase kinase 1/2; NIH3T3: mouse embryonic fibroblasts; NPCs: neural progenitor cells; NSCs: neural stem cells; PD: Parkinson's disease; PTCH1: Patched 1; HUWE: HECT, UBA, and WWE domain containing E3 ubiquitin protein ligase; SD: Sprague-Dawley; SHH: Sonic Hedgehog; SHH-MB: Sonic Hedgehog medulloblastomas; SUFU: suppressor of fused; TBI: traumatic brain injury; TTBK2: tubulin tyrosine ligase family member 2.

Studies have documented improvements in depression-like behavioral outcomes in rat models of depression following the administration of SHH agonists, including Gami-Shinkwhanin (Park et al., 2018) and ropinirole (Fatima et al., 2020). Concurrently, nicotine (administered at 0.3 mg/kg) potentiates brain-derived neurotrophic factor expression by amplifying SHH and Wnt signaling cascades, eliciting neuroprotective actions and ameliorating cognitive impairment (Tayyab et al., 2019b). The hippocampus, a neurogenesis and neural responsiveness hub, demonstrate heightened levels of SHH and GLI1 proteins after neogenin treatment in mice. Activation of SHH-GLI1 signaling promotes the proliferation of NSCs and NPCs, ultimately enhancing neurogenesis and excitatory neurotransmission and exhibiting antidepressant properties (Sun et al., 2018).

Furthermore, leonurine from the traditional Chinese medicinal plant Herba Leonuri, activates the SHH-GLI signaling axis, which in turn modulates hippocampal neuroregeneration in chronic unpredictable mild stress rats by modulating intestinal microbiota compositions (Meng et al., 2023). In the dentate gyrus of the hippocampus of depressed rats, *Zuogui Jiangtang Jieyu* Decoction triggered SHH activation, leading to a marked enhancement in the self-renewal capacity of NSCs and subsequent alleviation of depressive-like behaviors (Yang et al., 2023).

Notably, miRNAs are important for PD by their capacity to modulate NSC proliferation and differentiation. MiR-210-5p orchestrates the differentiation of human induced pluripotent stem cells into midbrain dopaminergic neural progenitors by targeting SMAD4 and SUFU to suppress TGF-β and activate the SHH signaling pathway (Lyu et al., 2023), while miR-7 negatively modulates the proliferation of DA progenitor cells through inhibition of the Wnt/β-catenin signaling axis and promotes regulation of the SHH signaling, thereby fine-tuning the equilibrium between oligodendrocyte and DA neuronal lineage commitment (Adusumilli et al., 2020). Furthermore, microRNA-124 stimulates dopamine receptor expression and neuronal proliferation by activating the SHH signaling cascade (Wang et al., 2019b). With similar effects observed for astragaloside IV (Gao et al., 2018) and *Zhichan* Decoction (Wentao et al., 2011) in fostering NSC proliferation and directing their differentiation towards DA neurons. Purmorphamine activates SHH signaling, safeguarding dopaminergic neurons and mitigating inflammatory responses by modulating the PI3K/Akt signaling pathway (Shao et al., 2017).

With the development of traditional Chinese medicine, increasing evidence suggests that acupuncture and moxibustion (AM), as well as massage, also play a significant role in treating various diseases. Studies have found that AM can improve SHH and GLI1 expression in the injured spinal cord, which may be part of the potential mechanism of AM treatment, including restoring motor function, protecting neurons, and reducing neuronal apoptosis after SCI (Ding et al., 2022). Resveratrol, an emerging anti-scarring agent, has recently been shown to activate the SHH signaling pathway and augment the transcriptional activity of GLI-1. This holds the potential to enhance the prognosis of fibrotic scars resulting from SCI (Guo et al., 2018). In TBI, N-cis-2,6-dimethylpiperidinocarbonyl-L-gamma-methylleucyl-D-1 methoxycarbonyltryptophanyl-D-norleucinol, an antagonist, can notably enhance the expression of SHH, subsequently mitigating the disruption of the BBB (Michinaga et al., 2021). Furthermore, miR-153 activates the SHH signaling pathway, fostering angiogenesis, and thus is potentially a therapeutic agent for treating traumatic brain injury (Wang et al., 2019b). Epigallocatechin-3-gallate, the main polyphenol in green tea (Lee et al., 2021), was recently found to promote the proliferation of adult NPCs and is dependent on SHH signaling (Sergi, 2022; Parashar et al., 2024). Lycium barbarum polysaccharides enhance SHH signaling and promote GLI1 protein expression, further promoting neurogenesis, reducing Aβ levels, and improving cognitive function (Zhao et al., 2016).

Trametinib, a potent MEK inhibitor, suppresses the activity of MEK1/2, thereby inhibiting the phosphorylation of ERK1/2. This leads to a significant downregulation of Gli1 and Gli2 gene expression, ultimately reducing the proliferation and viability of SHH-MB cells (Borlase et al., 2023). DS-1-038, (2-ethyl-5-(1-methyl-1H-pyrazol-4-yl) benzofuran-3-yl) (4-hydroxyphenyl) methanone, a derivative of benzarone, inhibits the phosphatase activity of EYA transcriptional coactivator and phosphatase 1, thereby reducing the expression and activity of GLI1. This inhibition downstream of the SHH signaling pathway affects the proliferation and survival of tumor cells (Hwang et al., 2024). In addition, compounds such as curcumin analogue BDDD-721, arsenic trioxide, and metformin also exert their effects by inhibiting the activity of key components in the SHH pathway, such as GLI1, thereby suppressing the migration, invasion, proliferation, and survival of SHH-MB cells (Dos Santos Klinger et al., 2020; Fang et al., 2022; Gong et al., 2022). Vismodegib, a potent MEK inhibitor, exerts its therapeutic effect on SHH-MB by allosterically inhibiting SMO. While vismodegib may lead to premature fusion of growth plates and resistance in pediatric patients (Robinson et al., 2017), recent findings suggest that direct intraventricular administration enhances therapeutic efficacy while avoiding systemic side effects, particularly in pediatric patients with SHH-MB (Kresbach et al., 2024). Furthermore, the HECT, UBA, and WWE domain-containing E3 ubiquitin ligase is an E3 ubiquitin ligase regulating PC disassembly by promoting the degradation of Tau tubulin kinase 2, reducing the proliferation of SHH-MB cells and triggering the differentiation of GNPs, which contributes to the treatment of SHH-MB (Lin et al., 2024).

Recent research highlights the therapeutic potential of the SHH signaling pathway in stroke treatment. Guanxin Ning Injection, a traditional Chinese medicine, has been shown to mitigate ischemia-reperfusion injury and reduce cerebral infarct volume by activating the SHH-PTCH1-GLI1 signaling pathway (Xiao et al., 2021). Another study reveals that resveratrol administration elevates SHH and Gli1 protein levels, stimulating Gli1 nuclear translocation (Yu et al., 2021). This enhances the proliferation, differentiation, and migration of NSCs/NPCs, while simultaneously suppressing astrocyte and microglia activation, resulting in reduced neuroinflammation. Experimental evidence indicates that SAG administration improves stroke outcomes by reducing astrocyte activation and dampening neuroinflammation (Nguyen et al., 2021). For cognitive deficits following stroke, Total saponins from *Trillium tschonoskii Maxim*. activate the SHH signaling pathway, increasing Nissl body density and dendritic spine formation, promoting synaptic remodeling and cognitive recovery (Wang et al., 2023a). Taurine supplementation similarly influences the SHH pathway to stabilize mitochondrial membrane potential, enhance ATP production, and increase mitochondrial DNA content, supporting mitochondrial function (Jia et al., 2023). These mitochondrial protective effects contribute to axonal sprouting and functional neurological recovery following ischemic stroke. The core function of these drugs is to activate the SHH pathway and provide a treatment method for stroke by reducing brain tissue loss, alleviating neuroinflammation, and maintaining synaptic plasticity.

## Tools and Techniques for Investigating the Sonic Hedgehog Signaling Pathway

### Application of gene editing technology in sonic hedgehog signaling pathway research

Gene editing is an advanced biotechnological approach that aims to manipulate gene expression or gene sequences to achieve specific biological functions or therapeutic effects. This technology involves the editing, regulation, or modification of genes to alter gene expression patterns, metabolic pathways, or phenotypes of cells or organisms (Garg et al., 2025). The primary objective of gene editing is to precisely control the activation, deactivation, or modification of genes, thereby regulating cellular behavior, disease treatment, or biological traits (Li and Brakebusch, 2024).

Among the various gene editing technologies, Zinc Finger Nucleases, Transcription Activator-Like Effector Nucleases, and CRISPR-Cas9 are the most prominent. The CRISPR-Cas9 system has emerged as the most widely used due to its high specificity, efficiency, programmability, and versatility (Razavi et al., 2024; Yun et al., 2024). The CRISPR/Cas9 system comprises the Cas9 protein, CRISPR RNA, trans-activating crRNA, single-guide RNA, and the protospacer adjacent motif. It functions through stages of recognition and guidance, DNA cleavage, cellular repair, and gene regulation, enabling precise genome editing (Wang et al., 2022d; Hussen et al., 2023).

CRISPR/Cas9 technology has revolutionized the study of the SHH signaling pathway by enabling precise gene editing, such as the rapid generation of gene knockout and knock-in models. It has also revealed the bidirectional regulatory function of SAFB-like transcription. SAFB-like transcription modulators act as co-inhibitors in the presence of GLI3 and as co-activators in the absence or low levels of GLI3, thereby regulating the expression of SHH target genes (Zhang et al., 2019). Furthermore, CRISPR/Cas9 has been used to mutate leucine 116 in the mouse *SMO* gene (corresponding to leucine 112 in the human SMO gene) to alanine. This mutation prevents cortisol from inhibiting the activation of SMO, thereby highlighting the key role of cortisol in regulating the SHH signaling pathway (Lu et al., 2025). Interestingly, enhanced autophagic activity was found in both the Gli2 knockout NIH3T3 cell line and the cell model with truncated mutations in the PTCH1 CTD region (Hsiao et al., 2018; Caballero-Ruiz et al., 2023), contributing to our understanding of the interaction between the Hedgehog signaling pathway and autophagy. CRISPR/Cas9 can introduce specific point mutations, insertions, or deletions with precision, allowing for a more accurate simulation of disease-associated mutations in humans. For example, it has been used to mimic human ODA (Chassaing et al., 2016), human HPE (Hong et al., 2020), and cardiovascular diseases. Through *CRISPR/Cas9* gene knockout technology, it was found that mutations in the PTCH1 gene can lead to abnormal cardiovascular development, including heart malformations, vascular network defects, and blood leakage, ultimately resulting in the death of early juveniles (Liu et al., 2024). Although these studies do not pertain to the central nervous system, they provide valuable models for studying SHH in central nervous system diseases.

The CRISPR/Cas9 can also edit the endogenous genome by inserting reporter genes or deleting specific regulatory elements to study the role of these regulatory elements in the SHH signaling pathway. For instance, CRISPR/Cas9 was used to precisely edit the first intron region of the GLI1 gene in human embryonic stem cells, removing six highly conserved GLI binding sites (Galat et al., 2021). This impaired the differentiation of multiple lineages during stem cell differentiation. Genetic ablation experiments demonstrate that PTCH1 and SUFU removal activates PI3K/AKT/mTOR cascades, inducing marked phenotypic transformations (Klein et al., 2019). Intriguingly, the knockout of the SUFU gene revealed that SUFU not only regulates the GLI3 protein in the SHH signaling pathway, but also directly influences the timing and proportion of astrocyte differentiation (Spice et al., 2022). These findings underscore the importance of gene interactions and pathway cross talk in understanding the complex regulatory mechanisms of the SHH signaling pathway in neurodevelopment.

When integrated with genome-wide screening approaches, CRISPR/Cas9 accelerates the discovery of SHH-associated genetic factors. For example, the key therapeutic target DNA methyltransferase 1 of SHH-MB was identified in the CRISPR/Cas9 whole genome knockout screening. Inhibition of DNA methyltransferase 1 effectively suppresses the growth of SHH-MB and exhibits a synergistic effect when used in conjunction with SMO inhibitors (Tsiami et al., 2024). The CRISPR/Cas9 technology, with its highly efficient and precise gene editing capabilities, has profoundly transformed the study of the SHH signaling pathway.

### Applications and prospects of bioinformatics and computational biology in the sonic hedgehog signaling pathway

Bioinformatics is an interdisciplinary field that integrates knowledge and tools from biology, computer science, information engineering, mathematics, and statistics to manage and analyze biological data (Zhao et al., 2024). Computational biology emphasizes mathematical modeling, algorithm development, and computer simulations to study the internal mechanisms of biological systems (Mbareche et al., 2020). Therefore, the study of the SHH signaling pathway using computational biology and bioinformatics can be conducted at multiple levels, including gene expression analysis, protein interaction network construction, signaling pathway modeling, and exploration of disease-related mechanisms.

#### Protein–protein interaction networks and AlphaFold

Protein–protein interaction networks (PPIs) describe the intricate cellular networks established through direct or indirect interactions between proteins (He et al., 2023). The PPI network serves as a fundamental framework for intracellular biochemical processes and signaling (Wang et al., 2024). In the study of the pathogenesis of neural tube defects and neuroectodermal tumors, the PPI network was constructed through the STRING database, revealing SHH as a common gene associated with neural tube defects and neuroectodermal tumors. SHH was found to be differentially expressed in RA-induced neural tube defects in mouse and human GBM samples (Cao et al., 2022).

Developed by DeepMind, AlphaFold represents a groundbreaking artificial intelligence system for predicting protein conformations (Omran et al., 2022; Abramson et al., 2024). Recent benchmarking studies demonstrate that AlphaFold’s predictive performance not only sets new standards in computational biology but occasionally exceeds the resolution of traditional experimental approaches (Jumper et al., 2021). While the application of AlphaFold to the SHH signaling pathway has yet to be extensively documented, its demonstrated precision in structural modeling implies the considerable potential for characterizing molecular components within non-canonical SHH pathways. Detailed predictions could reveal critical information about protein interaction interfaces, functional motifs, and dynamic binding behaviors, the computational advancements promise to accelerate mechanistic investigations of alternative SHH signaling mechanisms.

#### Ingenuity pathway analysis

Ingenuity pathway analysis is a powerful bioinformatics platform that integrates various biological data types, including genomic, proteomic, and chemical interaction networks, to establish an extensive knowledge base (De Angelis et al., 2022). It provides multiple functions for investigation, such as causal inference, upstream regulatory analysis, and downstream impact assessment, which help researchers decipher complex biological systems and identify potential biomarkers and drug targets (Krämer et al., 2014). In the context of SHH signaling pathway-related medulloblastoma research, ingenuity pathway analysis is utilized to analyze discrepancies in RNA and microRNA expression profiles among tumors with distinct mutation types, such as PTCH1 and SUFU mutations. The analyses clarify how varying transcriptional profiles modulate SHH pathway activity and connected signaling cascades, ultimately highlighting candidate molecules for pharmaceutical intervention (Gershanov et al., 2021). This tool’s analytical depth makes it invaluable for uncovering complex biological mechanisms and advancing therapeutic discoveries of SHH.

#### Boolean networks

Boolean networks are mathematical models designed to simulate the dynamic behavior of complex systems. By simulating and analyzing these networks, researchers can elucidate the behavior patterns of systems under various conditions (Pušnik et al., 2022). Through computational exploration of these models, researchers gain valuable understanding of how systems respond to different environmental or internal stimuli. The human brain as a complex and dynamic system, has profound implications for the study of neurodegenerative diseases (Rivas-Santisteban et al., 2023). A recent study has demonstrated that Boolean network models can effectively simulate the dynamic behavior of the brain and reveal the emergence and stability of neural circuits associated with cognitive function (Bertacchini et al., 2022). This methodology presents innovative opportunities for deciphering the complex operational mechanisms of the brain while simultaneously advancing our comprehension of SHH signaling mechanisms in neural tissue.

#### ActivePathways and Pathintegrate

ActivePathways is a pathway enrichment analysis method that integrates multiple omics data to identify significantly enriched biological pathways across datasets. It discovers associated genes through statistical data fusion (Paczkowska et al., 2020). In contrast, PathIntegrate utilizes machine learning algorithms to transform molecular-level omics information into pathway-centric representations (Wieder et al., 2024). The framework enhances both data synthesis and biological interpretability of complex datasets. The collaboration between ActivePathways and PathIntegrate may be more beneficial for studying the changes in the SHH signaling pathway under different disease states.

## Limitations

This review has some limitations. Most studies included are derived from animal models, such as rats and mice, with a relative scarcity of clinical data. Future investigations should prioritize obtaining additional patient-derived data to better characterize SHH signaling mechanisms within the CNS. Our review did not encompass brain diseases related to viral infections human immunodeficiency virus, when discussing neurodegenerative diseases.

## Conclusion

While the core SHH signaling mechanism appears relatively straightforward, its extensive interactions with multiple pathways, including but not limited to TGFβ, KRAS, Wnt/β-catenin, and NOTCH systems, create considerable functional complexity. Because of this, these intricate interconnections enable the pathway to influence numerous critical processes within the CNS, including neuronal cell fate specification, axon guidance, synaptic transmission, inflammatory responses, and mitochondrial dysfunction. This further underlines its close connection with the CNS. When analyzing the effect of SHH signaling in the CNS from the perspective of disease, we find that SHH activation can cause different cascades in different CNS diseases, and even in the same CNS disease, there may be differences due to time and space, which stems from the pathway’s inherent dynamic nature and its capacity for multifaceted crosstalk with other signaling networks.

The molecular machinery underlying this pathway is complex, and the effect of SHH on mitochondrial function appears controversial. Activation of the SHH signaling pathway promotes mitochondrial quality, fission or fusion balance, and ATP release (He et al., 2017). However, SHH-GLI signaling drives mitochondrial fragmentation, and exposure to SHH in CGNP significantly decreases MMP and ATP production (Malhotra et al., 2016).

Owing to the concurrent influence of canonical and non-canonical SHH signaling pathways on the CNS, their combined effects are extensive. However, it is crucial to emphasize that the intricate complexity of the CNS transcends the regulation of any single molecular mechanism. Hence, harnessing the SHH signaling pathway for treating CNS diseases represents a promising, yet not exhaustive, therapeutic avenue. As research delves deeper into uncovering atypical pathway mechanisms, the significance of SHH signaling continues to expand, concomitantly fostering the development of novel potential drugs. Consequently, translating these findings into clinical applications has become a priority. Furthermore, with the continuous advancement of technology, such as CRISPR gene editing, single-cell sequencing, and the further application of AI technology, the study of SHH signaling pathway will become more refined and systematic. These technologies will help us better understand the regulatory mechanisms of SHH signaling pathways.

## Additional files:

***Additional Table 1:*** *SHH regulates several aspects of the central nervous system.*

***Additional Table 2:*** *Drugs for the treatment of CNS disease by targeting SHH signaling.*

## Data Availability

*All relevant data are within the paper and its Additional files*.
